# Simultaneous Determination and Investigation of Nine Fungicides in Fruits Using Diethylenetriamine-Functional Magnetic Core-Shell Polymer Modified Graphene Oxide as an Efficient Adsorbent Coupled to UPLC-HRMS

**DOI:** 10.3390/ijms18112333

**Published:** 2017-11-05

**Authors:** Ming-Li Ye, Yan Zhu

**Affiliations:** Department of Chemistry, Zhejiang University, Hangzhou 310028, China; zhuyan@zju.edu.cn

**Keywords:** diethylenetriamine-functional magnetic core-shell polymer modified graphene oxide (DETA-MPs-GO), magnetic solid-phase extraction (MSPE), synergistic adsorption mechanism, ultra-performance liquid chromatography-high resolution mass spectrometry (UPLC-HRMS), fungicides

## Abstract

In this study, diethylenetriamine-functional magnetic core-shell polymer modified graphene oxide (DETA-MPs-GO) was prepared via precipitation polymerization and amidation reaction, and it was characterized by transmission electron microscopy (TEM), Fourier-transformed infrared spectroscopy (FTIR), and X-ray diffractometer (XRD). Subsequently, a magnetic solid-phase extraction (MSPE) procedure was applied to the as-synthesized DETA-MPs-GO for the detection of nine fungicides in fruit samples, prior to ultra-performance liquid chromatography-high resolution mass spectrometry (UPLC-HRMS). The homogenized fruit samples, spiked with D-labelled internal standards, were firstly extracted by 5 mL of acetonitrile twice and then purified by DETA-MPs-GO adsorbents. The optimization of the adsorption and elution conditions of DETA-MPs-GO toward fungicides was carried out to attain a satisfactory adsorption performance and desorption efficiency. The adsorption mechanism was carefully investigated, and the results revealed that a synergistic adsorption mechanism, including hydrogen bond and a π–π stacking interaction, was confirmed. Moreover, the limits of quantitation (LOQs) of the proposed approach were in the range of 0.01 to 0.30 μg/kg under the optimum conditions. The average recoveries at three spiking levels were 84.9% to 105.2%, with relative standard deviations (RSDs) varying from 0.8% to 8.2% (*n* = 6). The developed method was successfully utilized for the screening and detection of fungicides in 81 fruit samples purchased from markets. A detailed survey was carried out about the concentration distribution, types of fungicides, and combined use of fungicides in different fruits.

## 1. Introduction

Fungicides are among the most extensively exploited pesticides to destroy or prevent the growth of many plant pathogenic fungi in modern agriculture. Generally, due to their curative, protective, and eradicant functions, which are resistant to a large variety of crop fungal diseases [1,2], carbendazim, thiophanate-methyl, pyrimethanil, metalaxyl, prochloraz, procymidone, dimethomorph, triadimefon, and difenoconazole are widely applied in the cultivation of fruit, vegetables, and other crops. However, commonly used fungicides are highly toxic, and many of them have been proved to enervate the liver function, change the urinary bladder structure, and decrease kidney weight [3]. Considering their significant toxicity and potential mutagenicity, fungicides have received lots of attention all over the world [4]. Therefore, it is very important to develop an accurate and sensitive detection methodology for the simultaneous measurement of commonly used fungicides in fruits samples.

The traditional analytical methods to test the fungicides in different fruits are usually based on liquid chromatography (LC) [5,6,7] and gas chromatography (GC) [8,9,10]. Multiple techniques have been employed for the quantitation of fungicides. The coupling of efficient LC or GC with mass spectrometry detector (LC-MS or GC-MS) had become a powerful tool for the separation of the target analytes and accurate qualitative analysis. Compared with GC-MS, LC-MS is more user friendly and is commonly applied for substances with low volatility and thermal instability [11,12]. With the increasing qualitative and quantitative requirements, a general low resolution mass spectrometer could not confirm the exact structure of (semi) unknown compounds. The development of a high resolution mass spectrometer (HRMS) provides an effective approach to solve the above problems. It was often used in peptides sequencing [13,14]; hence there is an increasing focus on and interest in investigating the quantification of small molecules [15,16,17]. The Orbitrap-based high resolution mass spectrometer (HRMS) is a very recent technique based on an Orbitrap mass spectrometer coalescing with the high resolution performance of the Orbitrap and the selectivity of the quadrupole [18]. This combination is a promising way to screen emerging pollutants in environmental and biological samples, which is combined with the high energy collision (HCD) cell. High quality MS and MS^2^ spectra could be obtained, which is beneficial for further structural identification. However, matrix interference was universal during the LC-MS analysis; thus extraction and enrichment with high efficiency are increasingly indispensable prior to the instrumental detection. The research on highly efficient extraction materials has drawn much attention [19,20,21,22].

Graphene oxide (GO), a derivate of graphene (G), can help to improve the adsorption of target compounds, owing to its unique structure. A large number of epoxide, hydroxyl, and carboxyl groups were distributed on the surface of a GO sheet, and it had a large, delocalized, π-conjugated structure that can lead to high adsorption efficiency and a strong affinity for compounds with hydrophobic properties or aromatic ring structures. This work has focused on the novel application of GO with magnetic solid phase extraction (MSPE). The introduction of magnetic composites into a GO sheet could combine the high adsorption efficiency of the GO sheet with convenience and fast magnetic separation. Recently, the preparation of magnetic GO composites with a convenient co-precipitation method or electrostatic interaction has been commonly researched [23,24]. However, the magnetic GO composites prepared with these methods showed weak correlated driving force or physical force and showed unsatisfactorily acid-resistant natures.

Considering the above problems, this paper engaged with enwrapping a polymeric shell outside the magnetic microsphere so that it can availably protect the magnetic microsphere from damage in harsh conditions. Subsequently, the synthesized core-shell diethylenetriamine-functional magnetic polymers (DETA-MPs) were covalently assembled to the GO sheet. The as-prepared DETA-MPs-GO revealed high adsorption efficiency toward nine fungicides, and it was applied as a MSPE adsorbent in the simultaneous determination of nine fungicides in fruit samples combined with ultra-performance liquid chromatography-high resolution mass spectrometry (UPLC-HRMS).

## 2. Results and Discussion

### 2.1. Synthesis and Characterization of DETA-MPs-GO

**Figure 1:**
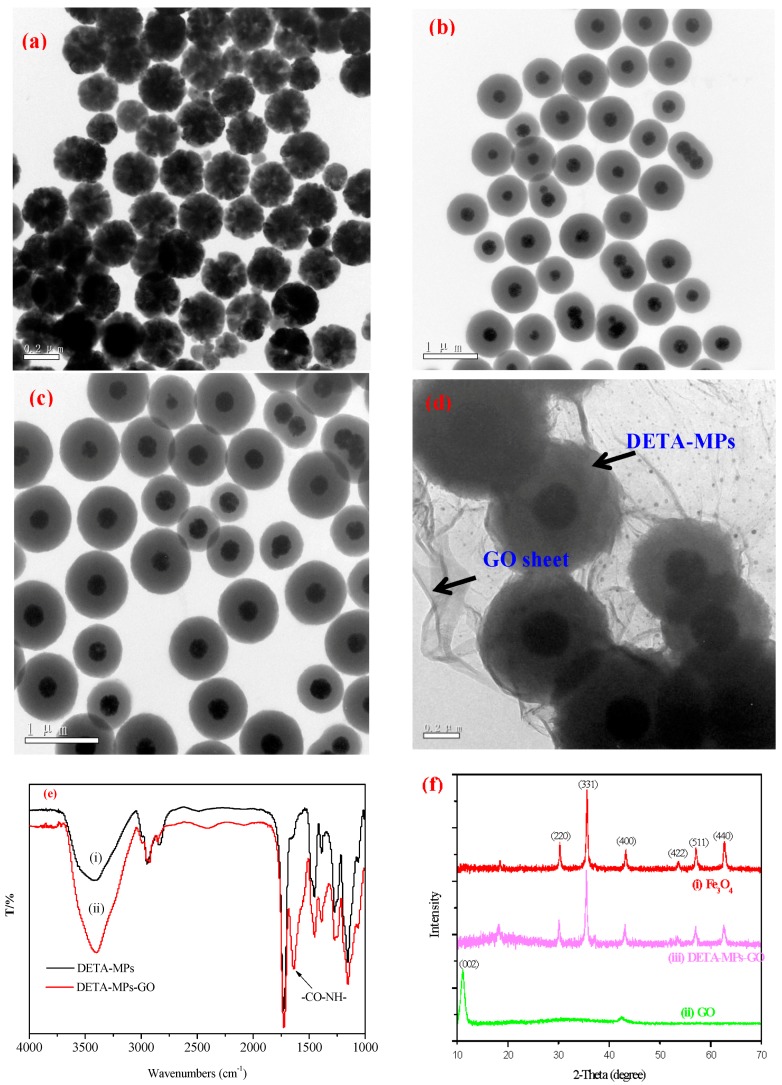
Transmission electron microscopy (TEM) images of: (**a**) magnetic Fe_3_O_4_ microspheres; (**b**) magnetic core-shell polymer (Fe_3_O_4_@poly(GMA-*co*-DVB)); (**c**) DETA-MPs; (**d**) DETA-MPs-GO; (**e**) Fourier-transformed infrared spectroscopy (FTIR); and (**f**) X-ray diffraction (XRD) spectra of synthesized materials. EDTA-MPs: diethylenetriamine-functional magnetic core-shell polymer, GO: graphene oxide.

Fe_3_O_4_ microspheres were prepared by solvothermal synthesis. It was observed that the synthesized Fe_3_O_4_ microspheres showed good spherical shape, with a coarse surface and narrow size distribution of around ~320 nm from the TEM photograph, as shown in Figure 1a. Then, well-defined core-shell Fe_3_O_4_@poly(GMA-*co*-DVB) was prepared via modified precipitation polymerization. In the polymerization process, DVB and GMA were chosen as two polymeric monomers since DVB showed a fast reaction rate, while GMA holds many epoxy groups. Furthermore, GMA was easy to locate outside the surface of the co-polymer microspheres in acetonitrile [25]. Hence, the amino groups were easily modified on the Fe_3_O_4_@poly(GMA-*co*-DVB) microspheres by a ring-opening reaction toward the outside reactive epoxy groups. As was observed in Figure 1b, the polymeric component was regularly coated outside the Fe_3_O_4_ core, and well-defined core-shell Fe_3_O_4_@poly(GMA-*co*-DVB) was achieved since the black core and gray shell could be clearly observed. Moreover, the Fe_3_O_4_@poly(GMA-*co*-DVB) also had a spherical shape but with a smooth surface. There is no significant difference between DETA-MPs and Fe_3_O_4_@poly(GMA-*co*-DVB) in terms of shape (Figure 1c). Figure 1d listed the last DETA-MPs-GO material, where DETA-MPs were immobilized on the GO sheet.

FTIR spectra were also conducted to further validate the success of grafting DETA-MPs onto the GO sheet. As could be observed in Figure 1e, the characteristic absorption of -NH_2_ groups occurred at ~3425 cm^−1^. Compared with DETA-MPs, a new characteristic absorption at ~1638 cm^−1^ arose after linking to the GO sheet, implying that DETA-MPs was covalently modified on the GO sheet through an amidation reaction. Finally, the XRD technique was also carried out to further demonstrate the structure and components of DETA-MPs-GO, and the as-synthesized Fe_3_O_4_, DETA-MPs and GO were tested, as exhibited in Figure 1f. It could be seen that the diffraction peaks at 2*θ* = 30.25°, 35.58°, 43.21°, 54.39°, 57.09°, and 62.92° could be owing to (220), (311), (400), (422), (511), and (440) reflections, respectively, confirming the cubic spinel crystal structure of Fe_3_O_4_. Figure 1f(ii) presents a sharp diffraction peak located at 2*θ* = 11.28°, which could be attributed to the (002) reflection of the GO sheet [26]. For DETA-MPs-GO (Figure 1f(iii)), the above six peaks of Fe_3_O_4_ could also been observed, but the (002) reflection absorption of the GO sheet could not be discovered. This was because the GO sheets can’t stack with each other anymore to form crystalline structures after being covered with magnetic polymer microspheres [27].

### 2.2. Optimization of UPLC-HRMS Conditions

The chromatographic conditions were carefully optimized to obtain the desired separation and retention on a column for the target analytes. Firstly, several experiments were carried out using different mobile phases, consisting of methanol or acetonitrile as the organic phase and water phase, respectively, with different proportions of formic acid in the range of 0.05% to 0.5%, as well as ammonium acetate and ammonium formic ranging from 1 mmol/L to 10 mmol/L. The results showed that methanol was more suitable since it was less polar and gave rather better chromatographic separation results than acetonitrile. Secondly, the addition of formic acid-ammonium acetate achieved much better results than that of formic acid-ammonium formic, since the ionization efficiency could be obviously improved. Finally, the optimal mobile phases were achieved using methanol as an organic solvent and water with 0.1% formic acid (*v*/*v*)-ammonium acetate (5 mmol/L).

**Figure 2:**
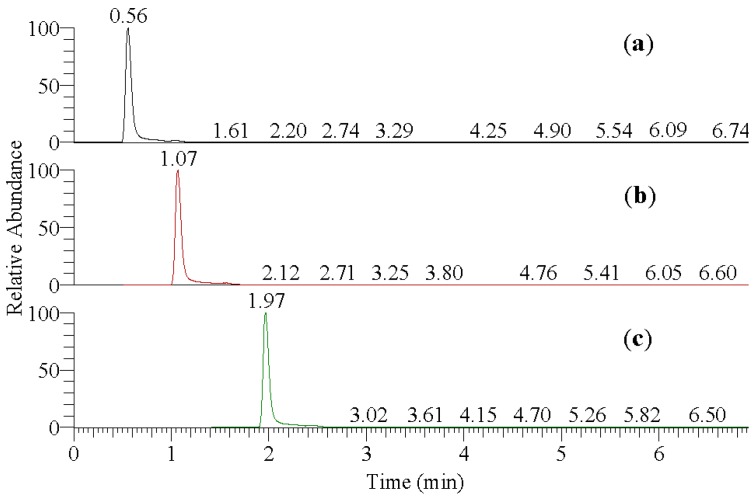
Selected ion monitor (SIM) chromatograms of carbendazim using different columns: (**a**) C_18_ column; (**b**) C_8_ column; and (**c**) Waters High Strength Silica (HSS) T3 column.

**Figure 3:**
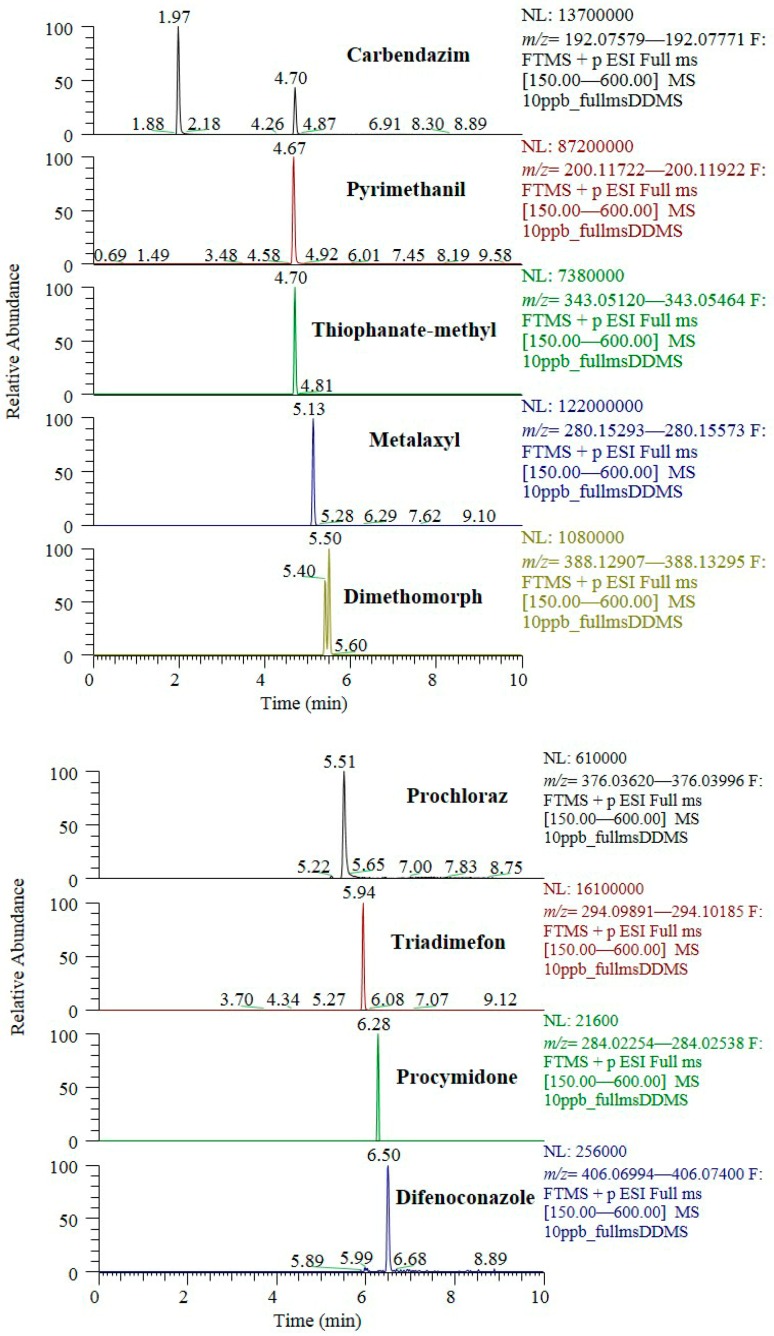
SIM chromatogram of nine target fungicides at 10.0 μg/L. NL: normalized level, FTMS: Fourier-transformed mass spectrometry, ESI: electrospray ionization, MS: mass spectrometry, DDMS: data-dependent mass spectrometry.

Furthermore, we selected several UPLC columns (C_8_, C_18_, Waters High Strength Silica (HSS) T_3_) to optimize the chromatographic retention of the nine target fungicides. Carbendazim, which was the most polar among the nine fungicides, was selected as the representative fungicide. As shown in Figure 2 carbendazim showed poor retention on the C_18_ column (*t* = 0.56 min, Figure 2a) because the structure of carbendazim was strongly polar. Better retention was obtained on the C_8_ column at 1.07 min (Figure 2b). However, satisfactory retention could be observed when using the Waters HSS T_3_ column (*t* = 1.97 min, Figure 2c), which can analyze polar structures. Therefore, Waters HSS T_3_ was selected as the separation column for the analysis of the nine fungicides. The typical selected ion monitoring (SIM) chromatogram of the nine target fungicides was shown in Figure 3, and it could be observed that their peaks were symmetrical and well separated.

The optimization of the HRMS conditions for the qualitative and quantitative analysis of the nine fungicides and their IS was achieved by determining the analytes using flow injection analysis. The sensitivity of the nine fungicides was verified by recording the chromatogram peak areas in a full mass scan under the positive ionization mode. The optimal UPLC-HRMS conditions for the nine fungicides were listed in Table 1. In Full MS/dd-MS^2^ mode, a Full MS scan was firstly carried out, and then a dd-MS^2^ scan was triggered, wherein the ions designated in the inclusion list or ions strong enough under the Full MS mode were chosen by quadrupole. Further, 1.0 Da was selected as an isolation width for the quadrupole. Furthermore, *N* corresponds to the loop count (*N* = multiplex × loop count, multiplex was set as five). Hence, the dd-MS^2^ spectra of the analytes only in Top 5 were carried out in the inclusion list with a mass deviation of less than 5 ppm at a certain retention time and above the set threshold. As shown in Table 1, the mass deviations of the nine fungicides were all less than 3.0 ppm.

### 2.3. Selection of Extraction Solvent

**Figure 4:**
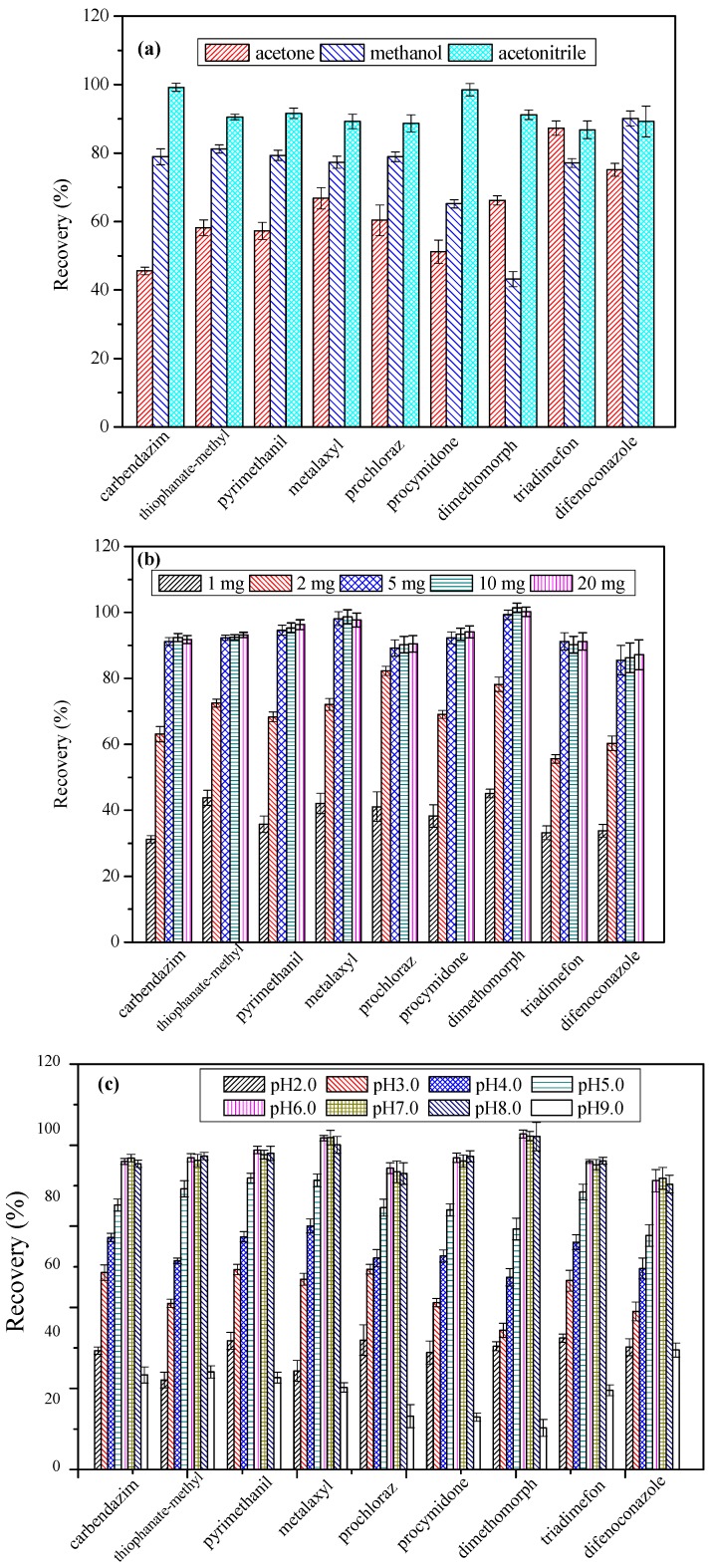

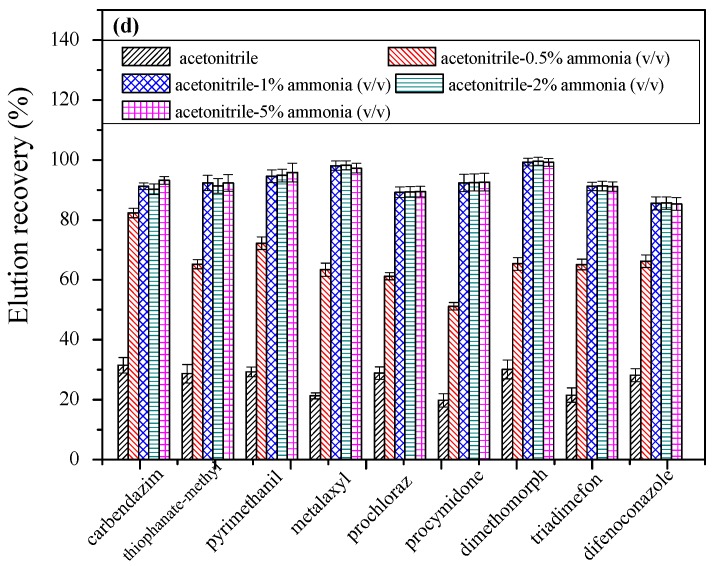
Extraction and desorption parameter optimization; each experiment was performed in sextuplicates: (**a**) extraction solvent; (**b**) adsorbent usage amount; (**c**) pH effect; (**d**) desorption solvent.

The selection of the extraction solvent was the most important step for sample preparation. It usually affected the qualitative and quantitative results of target analytes. Based on the “like dissolves like” principle, acetone, methanol, and acetonitrile were chosen as extraction solvents. The impact of the extract solvent on the extraction efficiency was studied by spiking nine fungicides in blank fruit samples (apple was selected as an example) at 5.0 μg/kg. The extraction recoveries for these extract solvents were determined, and the results shown in Figure 4a indicated that poor extraction recoveries were obtained by applying methanol or acetone as the extraction solvent for most of the nine fungicides, while acetonitrile gave excellent efficiencies. The possible reason was that the polarity of acetonitrile is closer to that of the nine studied fungicides than to that of acetone and methanol. Therefore, acetonitrile was selected as the extraction solvent in this study. Subsequently, the MSPE procedure with DETA-MPs-GO as an adsorbent was processed for the following purification.

### 2.4. Optimization of the Magnetic Solid-Phase Extraction (MSPE) Procedure

#### 2.4.1. Optimization of Adsorbent Amount Usage

The effect of usage amounts of DETA-MPs-GO on adsorption ability was measured using the blank sample extracts spiking with nine fungicides at 5.0 μg/kg. The spiking sample extracts were investigated by adding different usage amounts of DETA-MPs-GO, as shown in Figure 4b. It indicated that a significant influence could be seen on the adsorption efficiency of the fungicides due to the usage amount of DETA-MPs-GO adsorbent, and it was not difficult to observe a clear trend in the adsorption efficiency as the usage amount of DETA-MPs-GO ranged from 1 mg to 20 mg. When adding 1 mg or 2 mg of DETA-MPs-GO for the adsorption procedure, low recoveries of the nine fungicides were achieved, ranging from 31.2% to 82.3%. To increase DETA-MPs-GO, varying from 5 to 20 mg, the desired adsorption recoveries of the nine fungicides were achieved in the range of 85.5% to 101.4%. It could be easily discussed that the least usage amount (5 mg) of DETA-MPs-GO could availably concentrate the nine fungicides from fruit sample extracts.

#### 2.4.2. Optimization of Solution pH and Adsorption Mechanism Investigation

In this study, the pH effect was measured by applying the fruit extracts spiked with nine fungicides at 5.0 μg·kg^−1^ over the pH region of 2.0 to 9.0. As shown in Figure 4c, it was easy to observe that the optimal adsorption efficiency for the nine fungicides was obtained under pH 6.0 to 8.0. To increase or decrease the solutions’ pH values, the adsorption efficiency of DETA-MPs-GO toward fungicides decreased. This phenomenon could be explained by a synergistic adsorption mechanism. Firstly, the structures of the nine fungicides all have a benzene ring; hence π–π stacking would occur between the fungicide molecule and the GO sheet of DETA-MPs-GO. The adsorption behavior could be observed all over the pH region (Figure 4c). Secondly, all the fungicide molecules have nitrogen or oxygen atoms, which easily form hydrogen bonds with -NH_2_ (-NH-) groups in DETA-MPs-GO (including -O···H-N- and -N···H-N-). However, when the solution’s pH is less than 6.0, the main formation of the amino groups in DETA-MPs-GO is positive -NH_3_^+^, while N and O atoms in fungicide molecules are also easy to protonate. Therefore, electrostatic repulsion easily occurs between positive adsorbents and fungicides, leading to poor adsorption recoveries toward target fungicides at pH is less than 6.0. When pH is greater than 8.0, alkaline conditions could easily destroy the formation of hydrogen bonds, also resulting in low adsorption recoveries. When 6.0 ≤ pH ≤ 8.0, the main formation of an amino group on DETA-MPs-GO adsorbent is -NH_2_, and it was beneficial for the adsorption of target fungicides via hydrogen bonds. In summary, a solution pH of 7.0 was chosen for subsequent tests.

#### 2.4.3. Optimization of Elution Procedure

For the MSPE method, it was crucial to choose an effective desorption solvent for target compounds. Hence, in this study, the elution of nine fungicides was investigated carefully using several types of elution solvents, including pure acetonitrile and a series of acetonitrile solutions containing different proportions of ammonium hydroxide (*v*/*v*). The obtained results in Figure 4d indicated that pure acetonitrile was not very suitable for the desorption of nine fungicides, owing to the terrible desorption efficiency of the target fungicides (19.8% to 31.5%). It could be concluded that some other interactions took place, except a π–π interaction between fungicides and DETA-MPs-GO. The amino groups of DETA-MPs-GO were able to form stronger interactions with fungicides, for instance, hydrogen bonds. As shown in Figure 4d, alkaline acetonitrile performed better desorption recoveries than pure acetonitrile. This is because the hydrogen bond was easy to destroy under alkaline conditions. Considering the cost and convenience, acetonitrile containing 1% ammonium hydroxide showed the highest recovery; therefore it was chosen as the desorption solvent in subsequent tests.

The effects of the elution time and elution solution volume were also investigated. This revealed that 1.0 mL of acetonitrile containing 1% ammonium hydroxide was sufficient to desorb all nine fungicides, and 5 min was enough for the elution process to obtain the desired elution efficiency.

### 2.5. Matrix Effect

For food samples, matrix interference is a common phenomenon that may lead to either ion enhancement or the suppression of the target compound. For the purpose of decreasing the matrix effect, MSPE based cleanup was processed in a sample preparation combination of DETA-MPs-GO. The comparison between standards in neat solvent and spiked standards in three types of fruits (apple, orange, and grape) with a MSPE cleanup step showed a low matrix effect ranging from 86.4% to 99.0%, which is listed in Table 2. Therefore, it was necessary to process a MSPE cleanup with DETA-MPs-GO before UPLC-HRMS analysis, as described above. Nevertheless, little difference between the response standards in spiked fruits and in neat acetonitrile was still existent, ^13^C or D-labelled fungicides were applied as internal standards to reduce the matrix interference.

### 2.6. Method Validation

The concentrations used for the validation of the linear range were as follows: 0.1, 0.2, 0.5, 1.0, 5.0, 10.0, 20.0, and 100.0 μg/L for eight target fungicides, while 1.0, 2.0, 5.0, 10.0, 100.0, and 500.0 μg/L were used for for procymidone. 1.0 μg/L of nine internal standards, including d_4_-carbendazim, d_6_-thiophanate-methyl, d_5_-pyrimethanil, d_6_-metalaxyl, d_7_-prochloraz, d_8_-dimethomorph, d_4_-triadimefon, and d_6_-difenoconazole, were applied for quantification. Linear regression was performed on the ratios of the sample peak areas to the internal peak areas (Y) versus the mass concentration ratios (C, μg/L), with correlation coefficients (*R*^2^) above 0.9993 (Table 3).

The limits of detection (LODs) and limits of quantitation (LOQs) of the developed method for the nine target fungicides are shown in Table 3. The calculated LODs (S/N = 3) and LOQs (S/N = 10) were in the ranges of 0.01 to 0.30 μg·kg^−1^ and 0.03t o 0.90 μg·kg^−1^, respectively.

The tests of the precision and accuracy of the above method were carried out applying three blank typical fruit samples (apple, orange, and grape) spiked with nine fungicides at spiking concentrations of 1.0, 40.0, 80.0 μg/kg, respectively. The results are listed in Table 4. It was revealed that the mean recoveries of the nine fungicides ranged from 84.9% to 105.2%, with relative standard deviations (RSDs) in the range of 0.8% to 8.2%.

### 2.7. Application for Survey of Fungicides in Fruits Samples

**Figure 5:**
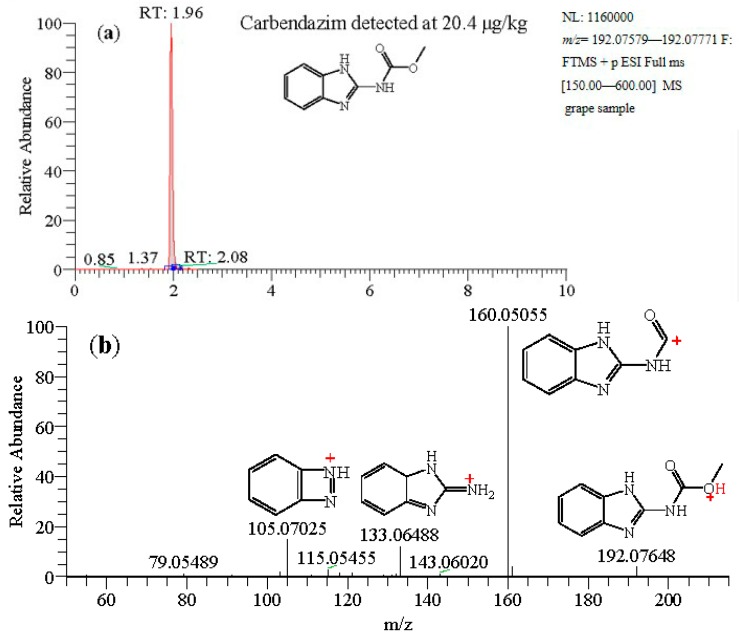
(**a**) Selected ion monitoring (SIM) chromatogram and (**b**) dd-MS^2^ spectra of a positive grape sample. RT: retention time.

The developed method was applied for the detection of fungicides in fruit samples bought from several supermarkets in China. A total of 81 fruit samples, including six types (grape, apple, pear, orange, yangtao, and watermelon), were detected, while 96.3% of them presented detectable levels of the target fungicides, the majority of which were carbendazim, with a positive frequency of 79.0% and a concentration region of 0.41 to 314.0 μg·kg^−1^ (Table 5). Figure 5 presented a positive grape sample that detected carbendazim at 20.4 μg/kg. It could be confirmed by dd-MS^2^ spectra, as shown in Figure 5b, that the main fragments were found to be *m/z* 160.05055, *m/z* 133.06488, and *m/z* 105.07025, owing to the fragments of [M+H-CH_3_OH]^+^, [M+H-CH_3_OH-CO]^+^, and [M+H-CH_3_OH-CO-C=N]^+^, respectively. Other fungicides such as thiophanate-methyl, pyrimethanil, metalaxyl, prochloraz, procymidone, dimethomorph, and difenoconazol were present in range of 3.7% to 49.4% of samples. The mean concentration of these varried from 1.2 μg·kg^−1^ to 287.7 μg·kg^−1^ (Table 5). Triadimefon could not be detected in all the analyzed fruits samples.

**Figure 6:**
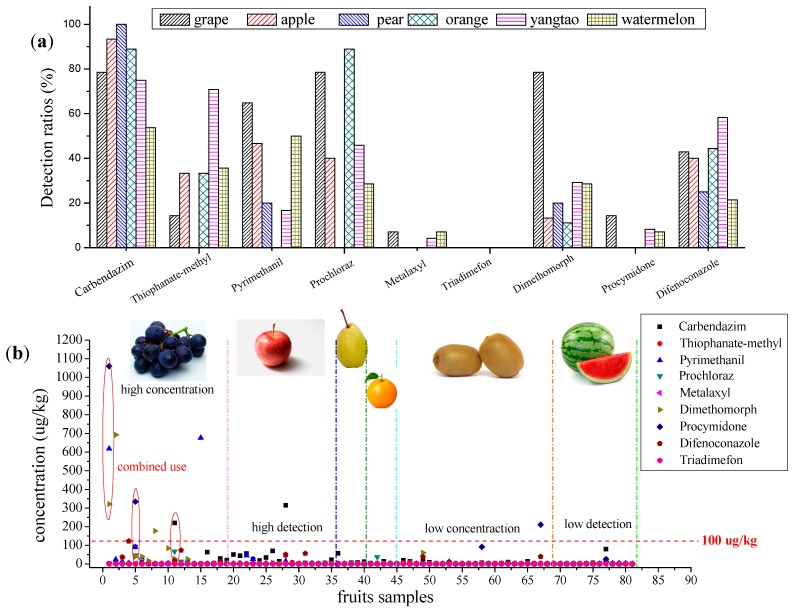
(**a**) Detection ratios of nine fungicides in 81 fruit samples; (**b**) detection concentrations of fungicides in 81 fruit samples.

As shown in Figure 6, for different varieties of fruits, the application of fungicides in grapes should be concerned with both the high detection ratio and high concentration result. Furthermore, the mixed use of the target fungicides in grapes is a universal phenomenon. For example, high concentrations of three types of fungicides such as 1060 μg/kg of procymidone, 618 μg/kg of pyrimethanil, and 321 μg/kg of dimethomorph were simultaneously detected in one grape sample. The main fungicide in apple samples was found to be carbendazim, the detection ratio of which was up to 93.3%. However, it was gratifying that most of them were at a low level (<100 μg/kg). In addition, lower concentrations of pyrimethanil and dimethomorph could also be detected in apple samples. Very small doses of fungicides, which were under 10 μg/kg, could be detected in pear samples. The levels of fungicides in orange and yangtao are both low; only procymidone was detected in one yangtao sample above 100 μg/kg. The types and dosages of fungicides in watermelon were the most satisfactory because all of them were lower than 60 μg/kg.

## 3. Materials and Methods

### 3.1. Reagents and Materials

The certified standards of carbendazim, thiophanate-methyl, pyrimethanil, metalaxyl, prochloraz, procymidone, dimethomorph, triadimefon, difenoconazole, d_4_-carbendazim, d_6_-thiophanate-methyl, d_5_-pyrimethanil, d_6_-metalaxyl, d_7_-prochloraz, d_8_-dimethomorph, d_4_-triadimefon, and d_6_-difenoconazole were supplied by Dr. Ehrenstorfer GmbH (Augsburg, Germany) with a purity of more than 98.0%. Analytical grade divinylbenzene (DVB), glycidyl methacrylate (GMA), and 2, 2-azobis (2-methyl-propionitrile) (AIBN) were supplied by J&K Chemical (Beijing, China). Diethylenetriamine and hydrochloric acid were purchased from Sinopharm Chemical Reagent Co., Ltd. (Shanghai, China). Ammonium acetate, formic acid, and acetonitrile were purchased from Merck KGaA (Darmstadt, Germany). All other chemicals were of analytical grade, unless stated otherwise.

### 3.2. Preparation of Diethylenetriamine-Functional Magnetic Core-Shell Polymer Modified Graphene Oxide (DETA-MPs-GO) Microspheres

**Scheme 1:**
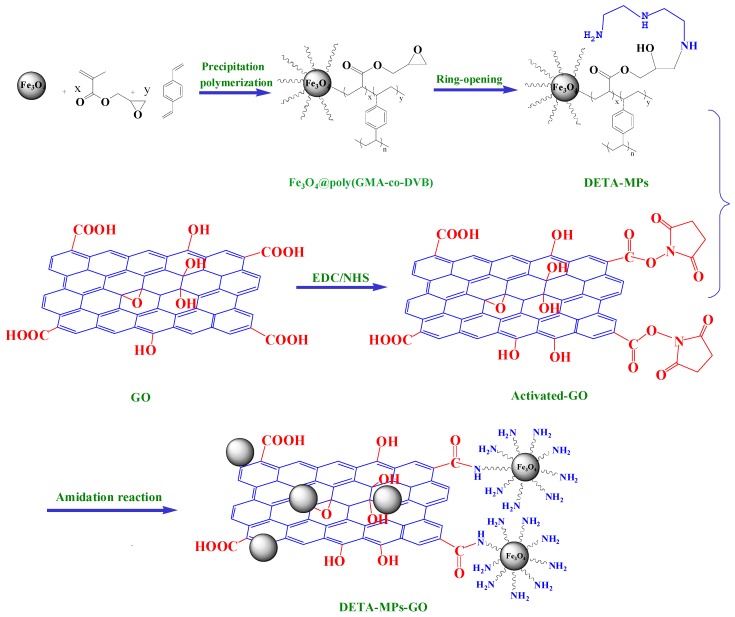
Synthesis process of diethylenetriamine-functional magnetic core-shell polymer modified graphene oxide (DETA-MPs-GO). GMA: glycidyl methacrylate, DVB: divinylbenzene, EDC: *N*-ethyl-*N*-(3-(dimethylamino)propyl)carbodiimide, NHS: *N*-hydroxysuccinimide.

The preparation of DETA-MPs-GO microspheres includes the following four steps, as shown in Scheme 1: (1) Magnetic Fe_3_O_4_ preparation: Magnetic Fe_3_O_4_ was prepared via a solvothermal approach, with a small modification of the literature [28]. In this procedure, 3.0 g FeCl_3_ was weighed into 80 g ethylene glycol (EG), the mixture was vortexed to achieve a yellow solution, and then 3.5 g NaAc and 2.0 g polyethylene glycol were added. Subsequently, the above solution was vigorously stirred for 40 min at room temperature to dissolve the residual solid and then transferred to a 150-mL autoclave, which was then reacted at 200 °C for 6 h. The magnetic Fe_3_O_4_ particles were achieved and cleaned using methanol to remove the adsorbed EG. (2) The synthesis of Fe_3_O_4_@poly(GMA-*co*-DVB): Fe_3_O_4_@poly(GMA-*co*-DVB) was prepared as follows: 10 mL GMA and 5 mL DVB were added into 40 mL acetonitrile, then the solution was stirred for 20 min to prepare a preassembly solution. The Fe_3_O_4_ microspheres (0.5 g) were then dispersed in 20 mL acetonitrile in a 150-mL round-bottomed flask and dispersed for 0.5 h under sonication. Then the preassembly solution, including GMA and DVB, was sneaked into the Fe_3_O_4_ suspension. Then the polymerization was carried out with thxe addition of 0.04 g 2, 2-azobis (2-methyl-propionitrile) (AIBN) under stirring at 600 rpm. The reaction solution was heated to 70 °C and maintained at 70 °C for 3 h. (3) Synthesis of DETA-MPs: the DETA-functional magnetic core-shell polymers (DETA-MPs) were synthesized via a ring-opening reaction between diethylenetriamine (DETA) and epoxide-functional Fe_3_O_4_@poly(GMA-*co*-DVB). In the process, 0.5 g of the achieved Fe_3_O_4_@poly(GMA-*co*-DVB) was firstly added into 100 mL methanol with 10 mL of dissolve DETA, and the mixture was stirred at 80 °C for 8 h. The resulting DETA-MPs was washed three times with methanol and dried in a vacuum oven at room temperature overnight. (4) Preparation of DETA-MPs-GO: DETA-MPs-GO was prepared under ultrasound conditions by an amidation reaction. 100 mg GO power in 50 mL water (2.0 mg/mL) was firstly dispersed under ultrasound for 2 h, and then 160 mg N-hydroxysuccinimide (NHS) and 200 mg N-ethyl-N′-(3-(dimethylamino)propyl)carbodiimide (EDC) were weighed into the above GO dispersion. The achieved dispersion was reacted under stirring for 1 h in order to activate carboxyl groups (-COOH) on the GO sheet. Afterwards, 500 mg DETA-MPs was introduced and dispersed under ultrasound for 20 min. The following reaction was performed at 40 °C for 4 h under vigorous stirring. The final DETA-MPs-GO was obtained after washing with methanol several times and dried for 12 h at 50 °C to remove any residual solvent.

### 3.3. Characterization of DETA-MPs-GO

Transmission electron microscopy (TEM, Hitachi H-7650, Kyoto, Japan) was used to test the morphology and dimensions of the DETA-MPs-GO composites, while Fourier-transformed infrared spectroscopy (FTIR) was recorded to measure the structure and composites of DETA-MPs on a FTIR spectrometer (NEXUS-470, Thermo Nicolet, Massachusetts, MA, USA). Furthermore, X-ray diffraction (XRD, Bruker D8 Advance) patterns were also carried out to determine the composites of the as-prepared materials.

### 3.4. Sample Extraction and Cleanup Procedure

About 2.0 g of comminuted fruit samples were weighed and added into a 50 mL polypropylene centrifuge tube. Then 10.0 μL of 100 μg/L internal standards (IS) solution was added to the tube. Subsequently, 5.0 mL of acetonitrile as extraction solvent was employed, and the sample solution was vortexed for 10 min after adding 1.0 g of sodium chloride and 1.0 g of magnesium sulfate. The sample was then centrifuged at 8000 r/min for 2 min. Afterwards the supernatant was transferred to another polypropylene centrifuge tube. The residues were repeat extracted with 5.0 mL of acetonitrile again. Subsequently, the two sample extracts were merged, 10 mL pure water was sneaked into the extract, the sample solution was adjusted to pH 7.0, and then 5.0 mg DETA-MPs-GO adsorbent was added for purification. The above mixture was further vortexed for 10 min. The magnetic adsorbent could be quickly collected under a magnet, and the supernatant was discarded. Subsequently, 1.0 mL of elution solvent was added to the elute target analytes by shaking for 5 min. The elution solution was similarly isolated in a magnetic field. Furthermore, a hydrochloric acid solution was used to adjust the elution solution to neutral, and then it was transferred to a 2.0 mL vial and evaporated to dryness under a N_2_ stream at 40 °C. Finally, the residue was re-dissolved with 0.5 mL acetonitrile and measured on UPLC-HRMS.

### 3.5. Ultra-Performance Liquid Chromatography Parameters

An UltiMate 3000 system (Thermo Scientific, Massachusetts, MA, USA) was used for separation, applying an Acquity HSS T_3_ column (2.1 mm ID × 100 mm, 1.8 μm) maintained at 30 °C. The analytes were separated by applying an aqueous solution containing 0.1% formic acid (*v/v*)-ammonium acetate (5 mmol/L) as eluent (A) and methanol as eluent (B). Gradient elution was performed as follows: 0.00 min~2.00 min, 10.0~40.0% (B); 2.00 min~7.00 min, 40~95.0% (B); 7.00 min~7.01 min, 95~10% (B); 7.01 min to 10.00 min, 10% (B). The flow rate was set to 0.30 mL/min, while 5.0 μL of sample solution was injected.

### 3.6. High-Resolution Mass Spectrometry Parameters

The detection of the target fungicides was performed on a high-resolution mass spectrometer (HRMS, Thermo Scientific, USA). A heated electrospray ionization source (HESI-II) was applied for the ionization of the nine fungicides in positive mode. The HRMS conditions were listed as follows: the ionization voltage was +3.5 kV; the sheath gas flow was 40 Arb; the auxiliary gas flow was 10 Arb; the capillary temperature was 320 °C; and the vaporizer temperature was 300 °C.

## 4. Conclusions

A rapid, accurate, and sensitive DETA-MPs-GO based MSPE-UPLC-HRMS method was established for the simultaneous detection of nine fungicides in fruit samples. The optimized conditions under an acidic LC gradient were proved to be effective at increasing the MS signals of fungicides. The pretreatment method based on MSPE with DETA-MPs-GO as an adsorbent offered the advantages of less matrix interference, high recovery, and greater precision. The successful application of the proposed method can be used as an efficient extraction and pre-concentration method for the determination of the presence of nine fungicides in 81 fruit samples.

## Figures and Tables

**Table 1:** The molecular formula, exact mass, mass deviation, and retention time of nine target fungicides.

Compounds	Retention time (min)	Molecular Formula	Ionization Mode	Theoretical Value of Exact Mass (Da)	Experiment Value of Exact Mass (Da)	Mass Deviation (ppm)
**Carbendazim**	1.97	C_9_H_9_N_3_O_2_	[M + H]^+^	192.07675	192.07632	−2.23
**Pyrimethanil**	4.67	C_12_H_13_N_3_	[M + H]^+^	200.11822	200.11786	−1.80
**Thiophanate-methyl**	4.70	C_12_H_14_N_4_O_4_S_2_	[M + H]^+^	343.05292	343.05240	−1.52
**Metalaxyl**	5.13	C_15_H_21_NO_4_	[M + H]^+^	280.15433	280.15393	−1.43
**Dimethomorph**	5.50	C_21_H_22_ClNO_4_	[M + H]^+^	388.13101	388.13052	−1.26
**Prochloraz**	5.51	C_15_H_16_Cl_3_N_3_O_2_	[M + H]^+^	376.03809	376.03702	−2.85
**Triadimefon**	5.94	C_14_H_16_ClN_3_O_2_	[M + H]^+^	294.10038	294.09973	−2.21
**Procymidone**	6.28	C_13_H_11_Cl_2_NO_2_	[M + H]^+^	284.02396	284.02338	−2.04
**Difenoconazole**	6.50	C_19_H_17_Cl_2_N_3_O_3_	[M + H]^+^	406.07197	406.07132	−1.60

**Table 2:** Absolute matrix effect, expressed as the ratio in percentage obtained with a spiked matrix versus neat solvent standards (1.0 μg/kg, *n* = 6).

Compound	Matrix	Average Peak Area		Absolute Matrix Effect (B/A) (%)
		Standard Solution (A)	Post-Spiked (B)	
**Carbendazim**	apple	3.14 × 10^6^	3.01 × 10^6^	95.9
	orange		3.11 × 10^6^	99.0
	grape		2.97 × 10^6^	94.6
**Thiophanate-methyl**	apple	2.81 × 10^5^	2.61 × 10^5^	92.9
	orange		2.49 × 10^5^	88.6
	grape		2.53 × 10^5^	90.0
**Pyrimethanil**	apple	1.28 × 10^6^	1.19 × 10^6^	93.0
	orange		1.23 × 10^6^	96.1
	grape		1.18 × 10^6^	92.2
**Metalaxyl**	apple	2.26 × 10^6^	2.01 × 10^6^	88.9
	orange		1.98 × 10^6^	87.6
	grape		2.08 × 10^6^	92.0
**Prochloraz**	apple	1.45 × 10^6^	1.37 × 10^6^	94.5
	orange		1.33× 10^6^	91.7
	grape		1.35 × 10^6^	93.1
**Procymidone**	apple	1.29 × 10^4^	1.20 × 10^4^	93.0
	orange		1.18 × 10^4^	91.5
	grape		1.23 × 10^4^	95.3
**Dimethomorph**	apple	2.94 × 10^5^	2.83 × 10^5^	96.3
	orange		2.85 × 10^5^	96.9
	grape		2.78 × 10^5^	94.6
**Triadimefon**	apple	8.25 × 10^5^	7.14 × 10^5^	86.6
	orange		7.19 × 10^5^	87.2
	grape		7.13 × 10^5^	86.4
**Difenoconazole**	apple	2.65 × 10^6^	2.42 × 10^6^	91.2
	orange		2.39 × 10^6^	90.3
	grape		2.44 × 10^6^	92.0

**Table 3:** Validation parameters obtained for the nine target fungicides in fruits.

Compounds	Linear Equation	Linearity Range (μg/L)	*R*^2^	LODs ^1^ (μg/kg)	LOQs ^2^ (μg/kg)
**Carbendazim**	Y = 1.01 C + 0.091	0.1–100.0	0.9996	0.03	0.09
**Thiophanate-methyl**	Y = 1.12C + 0.062	0.1–100.0	0.9994	0.06	0.18
**Pyrimethanil**	Y = 0.94C + 0.045	0.1–100.0	0.9998	0.01	0.09
**Metalaxyl**	Y = 0.98C + 0013	0.1–100.0	0.9999	0.06	0.18
**Prochloraz**	Y = 0.85C + 0.126	0.5–100.0	0.9996	0.10	0.30
**Procymidone**	Y = 1.05C + 0.315	1.0–500.0	0.9995	0.30	0.90
**Dimethomorph**	Y = 0.81C − 0.094	0.1–100.0	0.9993	0.03	0.09
**Triadimefon**	Y = 0.99C − 0.003	0.1–100.0	0.9995	0.01	0.03
**Difenoconazole**	Y = 1.12C + 0.211	0.5–100.0	0.9996	0.10	0.30

^1^ LODs: limits of detection, ^2^ LOQs: limits of quantitation.

**Table 4:** Accuracy and precision of nine fungicides in three blank representative fruit samples spiked at three different concentrations by the developed method (*n* = 6, $\bar{x}$ ± s).

Compounds	Average Recovery, % (RSD ^1^, %)								
	Apple			Orange			Grape		
	1.0 μg/kg	40.0 μg/kg	80.0 μg/kg	1.0 μg/kg	40.0 μg/kg	80.0 μg/kg	1.0 μg/kg	40.0 μg/kg	80.0 μg/kg
**Carbendazim**	96.6 (8.2)	105.2 (2.1)	98.1 (1.4)	90.1 (7.1)	93.2 (4.2)	96.4 (2.5)	96.2 (5.3)	102.3 (1.8)	99.3 (1.8)
**Thiophanate-methyl**	95.2 (5.3)	103.2 (1.2)	101.5 (0.9)	89.4 (3.5)	90.8 (2.3)	90.2 (2.0)	96.3 (3.2)	98.9 (1.0)	103.6 (0.8)
**Pyrimethanil**	90.2 (6.2)	94.5 (1.1)	96.2 (1.0)	87.1 (6.3)	91.2 (2.5)	94.7 (1.6)	92.5 (4.8)	96.2 (2.3)	98.7 (1.6)
**Metalaxyl**	95.3 (6.8)	96.7 (3.1)	97.5 (2.3)	93.4 (6.8)	95.3 (3.7)	95.8 (2.4)	94.2 (5.2)	97.8 (2.9)	99.1 (1.4)
**Prochloraz**	90.8 (6.9)	92.5 (2.6)	92.6 (1.8)	85.6 (6.2)	88.3 (2.8)	89.5 (1.9)	92.5 (4.7)	94.6 (3.2)	97.8 (1.9)
**Procymidone**	88.1 (7.2)	92.2 (3.2)	90.3 (2.1)	85.6 (7.8)	87.2 (4.2)	89.6 (3.1)	91.4 (6.2)	93.6 (2.8)	90.3 (1.6)
**Dimethomorph**	93.1 (5.2)	99.3 (4.2)	98.2 (2.4)	86.9 (6.4)	92.4 (4.0)	95.3 (3.5)	93.4 (5.4)	98.8 (3.6)	105.2 (2.1)
**Triadimefon**	86.3 (4.7)	89.6 (2.8)	90.2 (1.2)	85.6 (5.6)	89.5 (3.2)	91.3 (2.3)	86.5 (3.8)	89.8 (2.1)	92.6 (1.0)
**Difenoconazole**	85.2 (7.5)	88.1 (4.5)	91.2 (3.2)	84.9 (6.2)	86.7 (5.1)	93.5 (3.2)	89.7 (6.4)	89.5 (6.2)	93.3 (3.1)

^1^ RSD: relative standard deviation.

**Table 5:** Frequency, mean, minimum (min.), and maximum (max.) levels of the nine target fungicides detected in the analyzed fruit samples.

Compound	Frequency (%)	Concentration Detected (μg/kg)	
		Range (min.–max.)	Mean
**Carbendazim**	79.0	0.41–314.0	22.6
**Thiophanate-methyl**	39.5	0.32–45.2	3.35
**Pyrimethanil**	34.6	0.56–618	57.1
**Metalaxyl**	3.7	0.53–2.4	1.2
**Prochloraz**	49.4	0.40–693.0	56.9
**Procymidone**	6.2	6.17–1060	287.7
**Dimethomorph**	32.1	0.41–321.0	56.9
**Triadimefon**	0	-	-
**Difenoconazole**	42.0	0.42–121.6	13.3
